# Lenalidomide and the risk of serious infection in patients with multiple myeloma: a systematic review and meta-analysis

**DOI:** 10.18632/oncotarget.16235

**Published:** 2017-03-15

**Authors:** Li Ying, Tong YinHui, Zheng Yunliang, Haozhen Sun

**Affiliations:** ^1^ Department of Pharmacy, The First Affiliated Hospital of College of Medicine, Zhejiang University, Hangzhou, Zhejiang, P.R. China; ^2^ Department of Pharmacy, Zhejiang Cancer Hospital, Hangzhou, Zhejiang, P.R. China; ^3^ Research Center for Clinical Pharmacy, State Key Laboratory for Diagnosis and Treatment of Infectious Diseases, The First Affiliated Hospital of College of Medicine, Zhejiang University, Hangzhou, Zhejiang, P.R. China

**Keywords:** lenalidomide, multiple myeloma, incidence, infection, meta-analysis

## Abstract

The immunomodulatory drug lenalidomide is highly effective against newly diagnosed and relapsed/refractory multiple myeloma (MM), but serious and even fatal infections have been associated with its use. In this meta-analysis, we assessed the overall risk of infection to MM patients treated with lenalidomide. Eleven phase II or III clinical trials, comprising 3,210 subjects, were selected from the Embase, Pubmed, and Cochrane Library databases, from the Clinical Trial Registration website, and from meeting abstracts and virtual presentations at the American Society of Clinical Oncology. Main outcome measures were overall incidence, relative risk (RR), and 95% confidence intervals (CIs) of reported infection events. Fixed-effect or random-effect models were used in the statistical analyses, depending on the between-study heterogeneity. The overall incidence of high-grade infection was 14.32% (95% CI: 12.08%-16.90%) and high-grade infection's pooled RR was 2.23 (95% CI: 1.71-2.91, *P* < 0.0001) for all 11 studies evaluated. No evidence of publication bias for the incidence of high-grade infection was detected using Begg's funnel plot and Egger's test (*P* = 0.2; 95% CI: -1.70, 1.23). From this meta-analysis, it appears lenalidomide use is associated with an increased risk of high-grade infection. Moreover, fatal infection events occurred only in patients treated with lenalidomide; no infection-related deaths were observed among controls. These data indicate that accurate diagnosis and optimal management of infection in MM patients treated with lenalidomide could be critical for treatment efficacy.

## INTRODUCTION

Multiple myeloma (MM) is a hematological malignancy characterized by clonal proliferation of neoplastic plasma cells in the bone marrow (BM). The 10-year survival rate for patients with MM is approximately 30%, and more than 11,000 deaths occur every year in the United States due to this disease [1]. In combination with dexamethasone, the immunomodulatory drug lenalidomide has shown to improve clinical responses such as objective response rate, survival, and time to progression in patients with either newly diagnosed, relapsed, or refractory MM [2, 3] [4]. However, significant toxicity such as myelosuppression, neutropenia, thrombocytopenia, and moderate or serious infections, are commonly associated with its use. At present, it remains unclear to what extent treatment of MM with lenalidomide might be related to an increased risk of serious, potentially fatal, infections.

Lenalidomide is a structural analogue of thalidomide that exhibits much higher pharmacological activity [5]. The development of diverse infection events in patients receiving lenalidomide was reported in several clinical trials [6, 7]. As infection event data from many of such trials are rather limited, the difficulties that generally emerge from the analysis and interpretation of sparse adverse event data apply to evaluating the relevance of lenalidomide-related infections in MM therapy. As a consequence, the risk of serious or even fatal infection remains a concern, and hence closely monitoring infection symptoms is critically important upon initiation of lenalidomide therapy in patients with MM. When poorly managed, these may lead to fervescence, red swollen, ulceration with cave formation and even death. To assess the extent and magnitude of this problem, and to help guide treatment decisions, the incidence and relative risk of infection among MM patients receiving lenalidomide were evaluated in this meta-analysis.

## RESULTS

### Search results and trial characteristics

**Figure 1:**
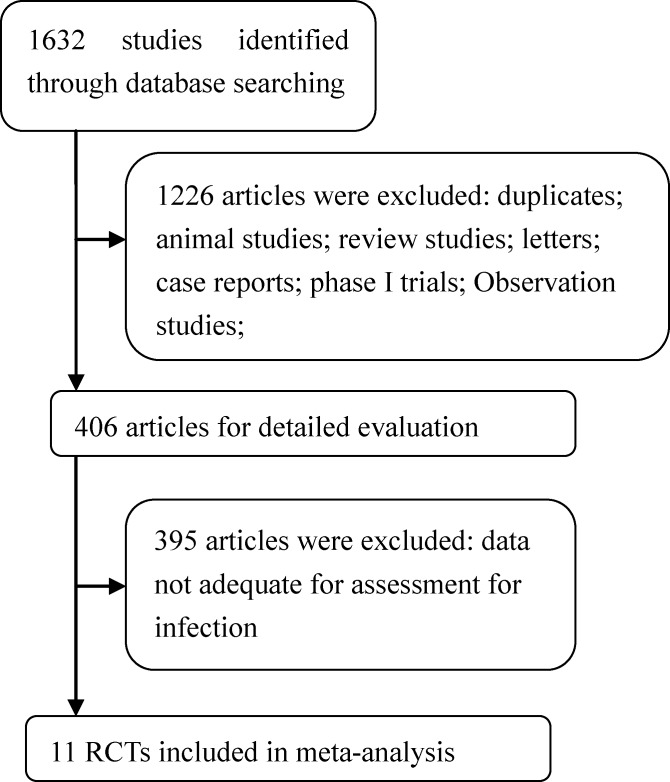
Flow chart demonstrating the process of study selection

Of the 1,632 potentially relevant studies yielded by our initial search, 1,621 were excluded per our review criteria (Figure 1). The remaining 11 studies [2–4, 6–13] included 3,210 subjects which met our inclusion criteria, and these were then available for analysis. Their characteristics are summarized in Table 1. The studies reviewed included three phase II trials [2, 4, 8] and eight phase III trials [3, 6, 7, 9–13], and all of subjects received lenalidomide and dexamethasone. Regarding study locations, six were from North America [4, 6–8, 10, 12], two from Greece [3, 9], and one each from France [11], Italy [13], and China [2]. The quality of the 11 studies was roughly evaluated in line with the Jadad scale: three studies had scores of 5 [7, 9, 11] four studies had scores of 4 [6, 10, 12, 13] and four studies had scores of 3 [2–4, 8] Score details for each study are shown in Table 1.

**Table 1:** Baseline characteristics of trials included in the meta-analysis (n=3210)

First author	Year	Trial phase	Ethnicity	Treatment	No.of HGI	No. of enrolled	Jadadscore
Rajkumar	2005	Phase II RCT	America	lenalidomide(25mg Daily) + dexamethasone	3	34	3
Dimopoulos	2007	Phase 3 RCT	Greece	lenalidomide (25mg Daily) plus dexamethasone	20	176	5
				Placebo + dexamethasone	11	175	
Jeffrey	2007	Phase 3 RCT	Greece	lenalidomide (25mg Daily) plus dexamethasone	14	100	3
				Placebo + dexamethasone	8	98	
Weber	2007	Phase 3 RCT	America	lenalidomide (25mg Daily) plus dexamethasone	38	177	5
				Placebo + dexamethasone	21	175	
Niesvizky	2008	Phase II RCT	America	lenalidomide(25mg Daily) + dexamethasone	5	72	3
Rajkumar	2010	Phase 3 RCT	America	Lenalidomide + high-dose dexamethasone	35	223	4
				Lenalidomide + low-dose dexamethasone	20	220	
Jeffrey	2010	Phase 3 RCT	America	lenalidomide (25mg Daily) plus dexamethasone	16	96	4
				Placebo + dexamethasone	11	94	
Attal	2012	Phase 3 RCT	France	lenalidomide (25mg Daily) plus dexamethasone	41	306	5
				Placebo + dexamethasone	15	302	
McCarthy	2012	Phase 3 RCT	America	lenalidomide (25mg Daily) plus dexamethasone	43	231	4
				Placebo + dexamethasone	14	229	
Palumbo	2012	Phase 3 RCT	Italy	lenalidomide (25mg Daily)	15	150	4
				Placebo	11	153	
Hou	2013	Phase II RCT	China	lenalidomide(25mg Daily) + dexamethasone	34	199	3

No. of HGI, Number of high grade infection events; No. of enrolled, Number of enrolled patients

This meta-analysis was performed in accordance with the guidelines of the Preferred Reporting Items for Systematic Reviews and Meta-Analyses (PRISMA) Statement (see the supplementary material in Appendix A1).

### Overall incidence of high-grade/fatal infection

**Figure 2:**
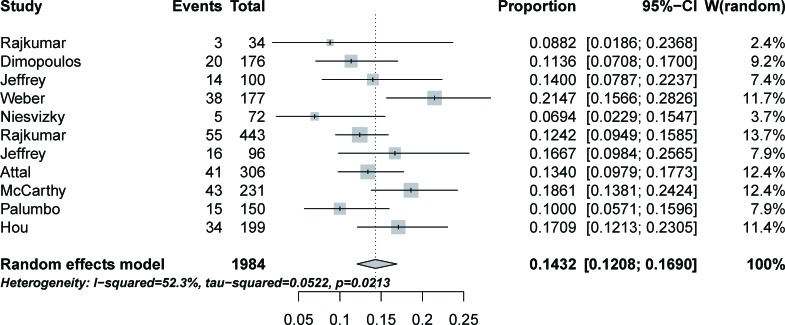
Forest plot for meta-analysis of incidence of high-grade infection in patients assigned lenalidomide

A total of 1,984 subjects from 11 studies [2–4, 6–13] were available for high-grade infection incidence analysis. High-grade infection events were reported in all studies, and incidence ranged from 6.94% to 21.47%. The highest incidence of infection was observed in a phase III trial in the US [7], in which all subjects were confirmed for progressive MM. Based on data from each study, the calculated overall incidence of high-grade infection was 14.32% (95% CI: 12.08%-16.90%) (Figure 2) according to the random-effects model (*P* = 0.02; I^2^ = 52.3%).

Fatal infection events were reported in two out of 11 studies, and incidence ranged from 0.43% to 1%. One occurred in a phase III trial from Greece [3], and the other in a phase III trial from the US [12]. Both fatal infection events occurred in patients treated with lenalidomide.

### Relative risk of high-grade/fatal infection

**Figure 3:**
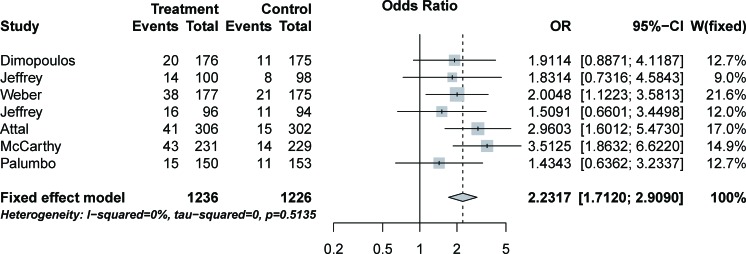
Relative risk of lenalidomide-associated high-grade infection

To evaluate the specific contribution of lenalidomide to the development of infection in MM patients, we evaluated the relative-risk (RR) of high-grade infection in lenalidomide and control groups after exclusion of confounding factors such as disease history and course. The 2,462 subjects from seven phase III trials [3, 7, 9–13] were included in the RR analysis. Treatment with lenalidomide significantly increased the risk of developing high-grade infection (pooled RR = 2.23; 95% CI: 1.71-2.91, *P* < 0.0001) (Figure 3), according to the fixed-effects model (*P* = 0.5135, I^2^ = 0%). The highest RR for any study was 3.51 (95% CI: 1.86-6.62) (Figure 3), and it was observed in a phase III study from the US [12].

### Publication bias

**Figure 4:**
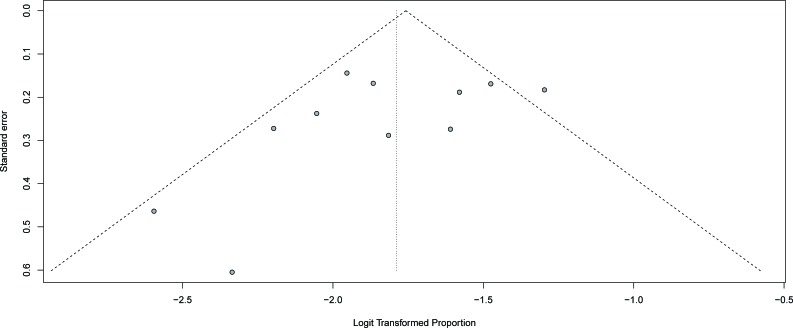
Funnel plot of the incidence of high-grade infection versus the study's standard error

No evidence of publication bias for the incidence of high-grade infection was found in our meta-analysis, as determined by funnel plot (Figure 4) and Egger's test (*P* = 0.2; 95% CI: -1.70-1.23).

## DISCUSSION

Infection is a major cause of mortality in patients with MM. Although stem cell transplantation and most novel anti-MM drugs increase the risk of infection, particularly during relapsed/refractory MM treatments, the risk posed by lenalidomide may be in this regard among the highest [14, 15]. Therefore, it is vital for the medical practitioner and patients to understand the risk of infection for optimization of treatment and management of this adverse event. Since available data is still scanty, we conducted this study to assess the risk and incidence of infection in MM patients receiving lenalidomide.

As far as we know, this is the largest meta-analysis to specifically assess the infection risk of lenalidomide in MM patients. We analyzed data from 3,210 patients with MM included in 11 clinical trials. The overall incidence of high-grade infection associated with lenalidomide in MM patients was 14.32% (highest incidence = 21.47%; lowest incidence = 6.94%). The pooled RR of developing high-grade infection was 2.23 (highest RR = 3.51). Fatal infection events occurred in two trials [3, 12] and only in patients treated with lenalidomide.

Usually, the incidence of infection is the highest in the initial months of drug treatment and in the last stage of the disease's progression [16, 17]; also, the incidence is lower in patients who have responded to treatment [14, 18]. Common clinical complications of MM patients that increase the risk of infection are neutropenia, neurodegenerative disease, kidney failure, fractures and other disease- and treatment-related comorbidities [19, 20]. Importantly, infection can lead to dose reduction or treatment discontinuation in the clinical management of MM. Our findings are significant in that that they indicate that the incidence of high-grade infection was twice as high in MM patients receiving lenalidomide than in controls. Therefore, preventive management and continued monitoring of infection symptoms are essential for MM patients receiving lenalidomide.

Lenalidomide-induced infections are difficult to accurately diagnose, and fever should be considered one of the manifestations of infection in MM patients until proved to be caused by other factors [21]. Examination of an infection traditionally starts with investigating the spectrum of causative agents in relation to current and past therapies and specific disease stages. The key to the optimal management of infection is using diagnostic tools to identify pathogens and applying antibacterial or antiviral treatment according to local epidemiological trends [15]. Otherwise, infection presents a clinical challenge for patients with MM, and the presence of various larvaceous pathogens is often persistent during the course of the disease [22]. Thus, all MM patients with lenalidomide-related infections should be carefully monitored and receive cautious care from physicians.

The mechanisms by which lenalidomide increases the risk of infection in MM patients remain unclear. While confounding factors may stem from age-related conditions, drug therapies, or the disease itself, MM-related immunodeficiency affects diverse cell types and immunological pathways, including B-lymphocyte dysfunction as well as functional abnormalities of natural killer cells, dendritic cells and T-lymphocytes [23, 24]. Lenalidomide-related organ dysfunction, such as digestive tract mucosa injury and lung and/or renal injury, may indirectly increase the incidence of infection [25]. Lastly, older patients suffering from chronic physical illnesses and senile diseases are more susceptible to infection, and the physician should be especially attentive to these patients.

Several limitations are present in our study. First, this is a meta-analysis based on previous studies, not on actual patient data. Thus, confounding variables, including basic medication history and adjuvant therapy, could not be considered in the analysis. Second, this meta-analysis was done using data from patients with proper organ functions, but the risk and prevalence of infection may be higher in routine clinical practice [26]. Third, the studies for this meta-analysis were performed at various types of institutions by different researchers, and the evaluations and conclusions may be heterogeneous [27].

In conclusion, our study showed that the incidence of high-grade infection was higher in MM patients treated with lenalidomide, and all fatal infection events occurred in this group. Adverse event monitoring is important to survey infections during lenalidomide treatment, and accurate diagnosis and optimal management of infection in MM patients is critical for safe medication prescription.

## MATERIALS AND METHODS

### Search strategy

We searched PubMed (from 1967), Embase (from 1974), and the Cochrane Library electronic databases through December 2016. Keywords included in the search were *lenalidomide*, *multiple myeloma*, *randomized controlled trials*, *clinical trials*, and *controlled clinical trials*. We also searched the Clinical Trials Registry website (ClinicalTrials.gov) to obtain information on registered clinical trials (RCTs). Additionally, we searched meeting abstracts and virtual presentations from the American Society of Clinical Oncology (ASCO; http://www.asco.org) up to 2015 for relevant RCTs. The search was restricted to clinical trials and articles published in the English language.

### Study selection and quality assessment

Two investigators (ZJ and LY) assessed the eligibility of the trials by independent search, and trials were retrieved for further consideration if they were judged pertinent by one or both investigators. Any discrepancies were identified and resolved by consensus. Clinical trials that met the following criteria were included:

a. Patients were diagnosed with MM.

b. Prospective phase II or III RCTs, including subjects assigned to treatment with lenalidomide.

c. Availability of data regarding events of infection.

The quality of all included trials was assessed using the Jadad scale, and scores ranged from 0 to 5, with a high score indicating a high quality study [28].

### Data extraction and clinical endpoints

Data extraction was performed by two investigators (ZJ and LY) independently, and infection data were extracted from the safety profile of all selected trials. For each trial, the following information was extracted: first author's name, year of publication, trial's phase, patient ethnicity, number of patients in the lenalidomide and control groups, and number of high-grade infection events. Treatment-emergent adverse events were recorded according to the Common Terminology Criteria for Adverse Events (CTCAE) of the National Cancer Institute (NCI).

### Data analysis

Our analysis was performed according to the Preferred Reporting Items for Systematic Reviews and Meta-Analyses (PRISMA) statement [29]. The principal indices were incidence, relative risk (RR), and corresponding 95% CIs of relevant infection events. To calculate incidence, the number of subjects with high-grade (grades 3, 4 or 5) infection and the total number of subjects treated with lenalidomide were extracted from the safety profiles of the included trials. The proportion and 95% CI of subjects with infection were derived in each trial, and the RR of infection was derived only in trials with a control group in the same trial. For trials reporting zero events in any group, we applied a classic half-integer continuity correction to calculate the RR and variance [30]. Statistical heterogeneity was assessed by using Cochran's Q statistic [31], and inconsistency was quantified with I^2^ tests among the included trials [32]. Heterogeneity was considered statistically significant when *P* < 0.1 or I^2^ > 40%. If heterogeneity existed, the data was analyzed using a random-effects model; if heterogeneity did not exist, a fixed-effects model was used. A statistical test with a *P*-value less than 0.05 was considered significant. The presence of publication bias was estimated using the Begg's and Egger's tests [33, 34]. All data analyses were performed using R software, version 3.2.3 (The R Project for Statistical Computing, http://www.r-project.org).

## SUPPLEMENTARY MATERIALS FIGURES AND TABLES


